# Long‐read sequencing reveals common structural variants as potential drivers of genetic rick for neurodegenerative diseases

**DOI:** 10.1002/alz.092769

**Published:** 2025-01-03

**Authors:** Alex N Salazar, Niccoló Tesi, Daniel Alvarez Sirvent, Lydian Knoop, Yaran Zhang, Sven J van der Lee, Sanduni Wijesekera, Jana Krizova, Wiesje M. van der Flier, Yolande A.L. Pijnenburg, Marcel JT Reinders, Marc Hulsman, Henne Holstege

**Affiliations:** ^1^ Genomics of Neurodegenerative Diseases and Aging, Human Genetics, Vrije Universiteit Amsterdam, Amsterdam UMC, Amsterdam Netherlands; ^2^ Alzheimer Center Amsterdam, Neurology, Vrije Universiteit Amsterdam, Amsterdam UMC location VUmc, Amsterdam Netherlands; ^3^ Alzheimer Center Amsterdam, Neurology, Vrije Universiteit Amsterdam, Amsterdam UMC, Amsterdam Netherlands; ^4^ Delft Bioinformatics Lab, Delft University of Technology, Delft, Delft Netherlands

## Abstract

**Background:**

Genome‐Wide Association Studies (GWAS) have identified 86 SNPs associated with Alzheimer’s disease (AD). GWAS‐SNPs are markers of genetic variation in linkage disequilibrium (LD), which may drive the association with AD. One major class of genetic variation are Structural Variants (SVs), which can regulate transcription and translation of nearby genes. Here, we explored the interplay between large SVs (>50 bp) and AD‐associated SNPs.

**Method:**

We performed long‐read whole‐genome sequencing of 214 individuals representing the extreme ends of the cognitive spectrum: N = 93 AD patients (age 67.2∓8.5), and N = 121 cognitively healthy centenarians (age 101.2∓1.8). We identified SVs using *sniffles2* genome‐wide, further characterised them through (local) *de novo* assembly, and annotated tandem repeats (TR) and transposable elements (TE). Next, we estimated LD between SVs and 86 AD‐associated GWAS‐SNPs, Finally, we compared SNP frequencies and SV sizes between AD cases and centenarians using logistic regression models.

**Result:**

Across all 214 individuals, we found >27,404 SVs (>50 bp), with a Minor Allele Frequency (MAF) ≥ 5%. Most SVs were TRs (63%), followed by TEs (37%). We found that 37 AD GWAS‐SNPs paired with 90 SVs in low to strong LD (R^2^ > 0.1), 56 of which involved SNP‐TR pairs (62%) and 34 involved SNP‐TE pairs (38%). In six AD loci, the SVs associated more strongly with AD risk than the leading GWAS‐SNP (p < 0.05). This includes *ADAM10, CTSH, SLC2A4RG, CD2AP, SPI1*, and *IDUA*, which were encompassed by complex haplotypes harbouring multiple TRs and TEs.

**Conclusion:**

Our findings provide the first support for SVs as candidate‐drivers of the association between respective GWAS loci with AD risk. Hence, SVs may lead to increased effect sizes.

